# Plasma tissue factor coagulation activity in post-acute myocardial infarction patients

**DOI:** 10.3389/fendo.2022.1008329

**Published:** 2022-09-23

**Authors:** Xiong Chang Lim, Siti Maryam J. M. Yatim, Suet Yen Chong, Xiaoyuan Wang, Sock Hwee Tan, Xiaoxun Yang, Siew Pang Chan, A Mark Richards, Chris J. Charles, Mark Y. Chan, Jiong-Wei Wang

**Affiliations:** ^1^ Department of Surgery, Yong Loo Lin School of Medicine, National University of Singapore, Singapore, Singapore; ^2^ Cardiovascular Research Institute, National University Heart Centre Singapore, Singapore, Singapore; ^3^ Department of Medicine, Yong Loo Lin School of Medicine, National University of Singapore, Singapore, Singapore; ^4^ Nanomedicine Translational Research Programme, Centre for NanoMedicine, Yong Loo Lin School of Medicine, National University of Singapore, Singapore, Singapore; ^5^ Department of Physiology, Yong Loo Lin School of Medicine, National University of Singapore, Singapore, Singapore

**Keywords:** tissue factor activity, acute myocardial infarction, left ventricular remodeling, heart failure, coagulation

## Abstract

**Introduction:**

Coagulation is involved in fibroproliferative responses following acute myocardial infarction (AMI). Left ventricular (LV) remodeling following AMI is closely associated with progression to heart failure. This study aims to assess the association between plasma tissue factor activity and LV remodeling in post-AMI patients.

**Methods:**

We studied 228 patients with AMI and 57 healthy subjects. Patients with AMI were categorized into two age- and sex-matched groups: patients with adverse LV remodeling or reverse LV remodeling, defined by an increase or decrease, respectively, in LV end systolic volume by ≥15% over 6 months. TF activity was measured in plasma collected at baseline (within 72 hours of revascularization), 1 month and 6 months post-AMI. Multiple level longitudinal data analysis with structural equation (ML-SEM) model was used to assess the impact of various clinical variables on TF activity in post-AMI.

**Results:**

Plasma TF activity in post-AMI patients at baseline (29.05 ± 10.75 pM) was similar to that in healthy subjects but fell at 1 month (21.78 ± 8.23, p<0.001) with partial recovery by 6 months (25.84 ± 8.80, p<0.001) after AMI. Plasma TF activity at 6 month post-AMI was better restored in patients with reverse LV remodeling than those with adverse LV remodeling (27.35 ± 7.14 vs 24.34 ± 9.99; p=0.009) independent of gender, age and relevant cardiovascular risk factors.

**Conclusions:**

Plasma TF activity decreased after AMI but was better restored at 6 months in patients with reverse LV remodeling. The clinical significance of changes in post-AMI plasma TF activity needs further investigation.

## Introduction

Thrombus formation, due to atherosclerotic plaque disruption and exposure of subendothelial tissue factor (TF), is the key event underpinning atherothrombosis in acute myocardial infarction (AMI) (1). Following AMI, severe myocardial injury adverse left ventricular (LV) remodeling is associated with progression to heart failure. Remodeling is a complex process incorporating changes in ventricular shape, size and function secondary to haemodynamic stress, neurohormonal activation and pro-inflammatory cytokines triggered by the acute loss of myocytes (2). Interestingly, serum levels of interleukin-6 (IL-6) and TF are highly correlated in patients with heart failure (3, 4), suggesting a crosstalk between inflammation and coagulation during LV remodeling.

TF is a transmembrane protein that primarily functions as an initiator of the extrinsic coagulation cascade. It binds to coagulation factor VIIa, resulting in activation of coagulation factors IX and X, ultimately leading to fibrin formation (5, 6). Apart from its role in hemostasis, TF promotes inflammation and angiogenesis through different signal transduction pathways (6–9). TF is mainly expressed in perivascular tissue, circulating cells, and in blood as well as in the myocardium (5, 9, 10). Besides the presence of cellular TF, circulating TF is also detectable in extracellular vesicles in the bloodstream of healthy individuals (11). Cardiovascular risk factors such as hypertension, diabetes, dyslipidemia, hypercholesterolemia and smoking have been shown to increase levels of circulating TF (12–14). Conversely, some drugs such as angiotensin-converting-enzyme inhibitor (ACE inhibitor), HMG-CoA reductase inhibitors (statins) and anti-platelet agents are associated with decreased TF expression (12).

TF maintained in an encrypted state on the cell surfaces has very little procoagulant activity until it is activated (15, 16). Reported associations of plasma TF with adverse cardiac events and mortality post-AMI (17–23) are based on the measurement of TF antigen levels which do not fully reflect the functional capacity of TF (24). Moreover, there are few data on the association between plasma TF activity and LV dysfunction post-AMI. We therefore profiled temporal changes in plasma TF activity following AMI and evaluated their association with LV remodeling.

## Materials and methods

### Study design and population

**Figure 1:**
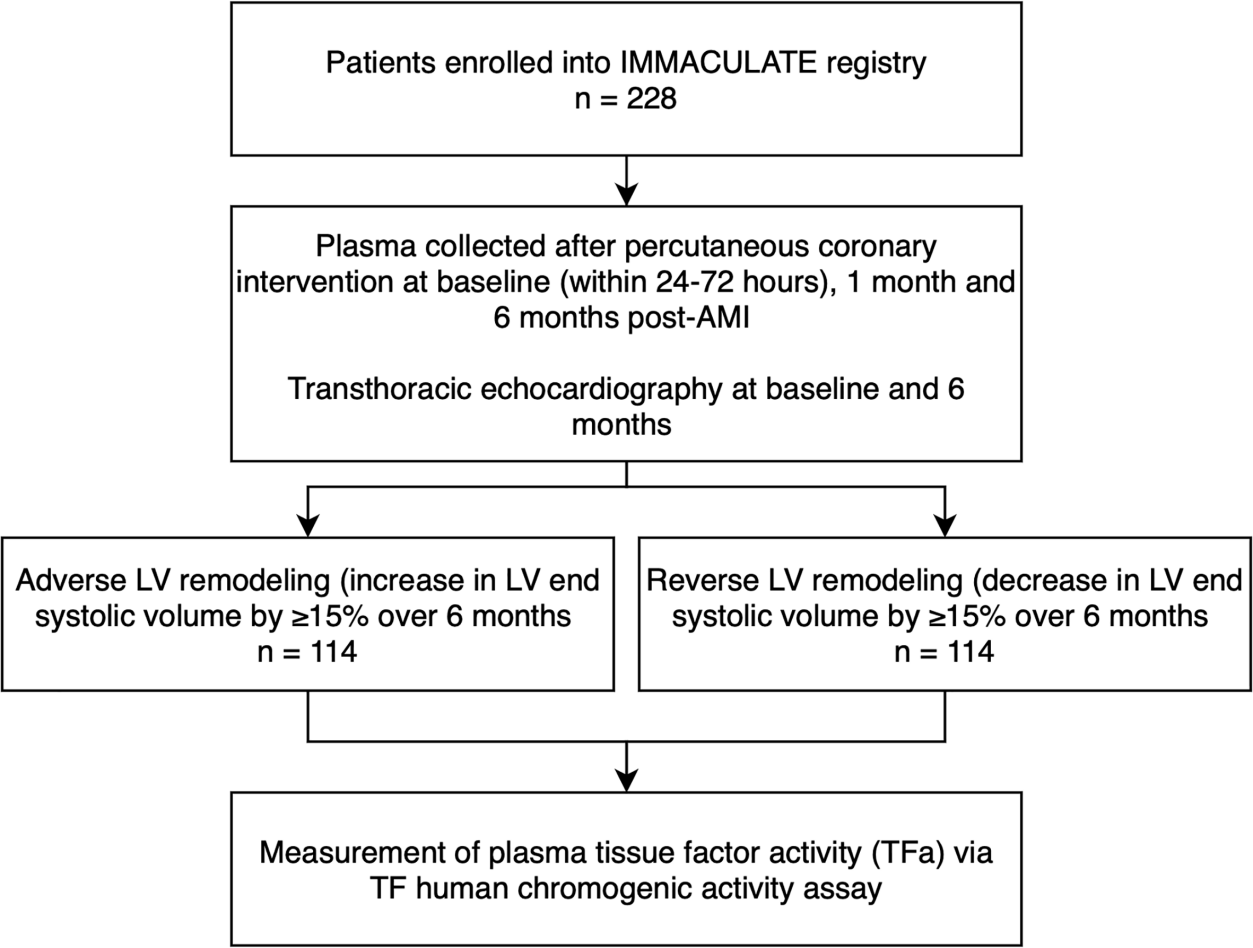
Study design flowchart.

The present analysis is a sub-study of the IMMACULATE registry study (**Figure 1**), a nested case-control study matching 114 patients with adverse LV remodeling to 114 patients with reverse LV remodeling who underwent urgent coronary angiography for percutaneous coronary intervention (PCI) (25). The IMMACULATE registry was a multicenter study enrolling patients hospitalized for AMI as established by the presence of ischemic chest pain or angina equivalent symptoms associated with electrocardiographic and cardiac enzymatic changes. Electrocardiographic changes included: an ST-segment elevation of two or more contiguous leads in leads V2 – V3 of at least 0.2 mV in in men or 0.15 mV in women, or an elevation of at least 0.1 mV in other contiguous chest leads, or the limb leads. Patients with clinically diagnosed non-ST-elevation MI (NSTEMI) established by the presence of electrocardiographic changes of ST-segment depression or prominent T-wave inversion and positive biomarkers of cardiac necrosis (troponin, CKMB) were also included in this study. Exclusions were age over 85 years, valvular heart disease, cardiogenic shock, malignancy, renal impairment (eGFR < 15 ml/min/1.73 m^2^), liver impairment, anemia, HIV, hepatitis B or hepatitis C. Clinical outcomes recorded at 1- and 6-months post-AMI after recruitment, included cardiovascular death, heart failure, recurrent MI, and ischemic stroke. At baseline and 6 months post-AMI, patients underwent transthoracic echocardiography for assessment of cardiac structure and function. Adverse LV remodeling was defined as an increase in left ventricular end systolic volume (LVESV) by ≥15% over 6 months and reverse LV remodeling as a decrease in LVESV by ≥15% over 6 months. Blood samples were collected at baseline (defined as within 24-72 hours of revascularization), and subsequently at 1 month and 6 months post-AMI. Samples were collected in 3.2% (w/v) sodium-citrate anticoagulated tubes and spun for 10 minutes at 4000g to separate plasma which was aliquoted and stored at -80°C until analysis.

A control group of 57 healthy subjects was recruited for this study. The healthy subjects comprised 45.6% males (n = 26) with a mean age of 35 years and 47 (82.5%) Chinese. All participants provided written informed consent. The study was approved by the National University of Singapore Institutional Review board (NHG DSRB Ref: 2015/01156) and conducted according to the principles of the Declaration of Helsinki.

### Plasma tissue factor activity assay

Plasma TF procoagulant activity was measured using the Tissue Factor Human Chromogenic Activity Assay Kit (ABCAM, #ab108906) as previously described (26). Briefly, 10 µL plasma was added to assay mix comprising of 50 µL assay diluent, 10 µL factor VII and 10 µL factor X and mixed. The mixed sample was incubated at 37°C for 30 minutes and subsequently 20 µL FXa substrate was added to the sample. Absorbance at 405 nm was measured every 5 minutes for 35 minutes with a microplate reader. Standard curves were prepared by serially diluting standard solution (500pM) 1:2 with sample diluent which correlated with TF activity.

### Data analysis

All statistical analyses were performed with SPSS version 25.0 (IBM Corp., Armonk, NY) and Stata MP 16.0 (Stata Corp., Texas, USA). Statistical graphs were generated using GraphPad Prism 7.00 (GraphPad Software, San Diego, California, USA). *P*<0.05 was considered statistically significant. No imputation of missing data was performed as the number of missing data for the respective variables (plasma TF activity at baseline = 2; diabetes = 1; smoker = 1; total cholesterol = 8; triglyceride = 7; high-density lipoprotein = 7; low-density lipoprotein = 10) was less than 5% of the sample size.

Continuous data were expressed as mean ± standard deviation (SD) or median ± interquartile range (IQR). Categorical variables were expressed as number with percentage (%). Differences between groups were analyzed with Chi-square test or unpaired Student’s t-test as appropriate. The differences between baseline and follow-up measurements were established using paired t-test. Plasma TF activity differences were tested in a univariable logistic regression model as well as in a multivariable model adjusted for known cardiovascular risk factors. Subsequently, the multilevel structural equation model (ML-SEM) was performed to ascertain the possible associations of clinical variables related to cardiovascular disease on plasma TF activity post-AM. The construction of this model takes into consideration the longitudinal design and the sequential nature of the data, thus allowing the accommodation of complex data interaction.

## Results

### Baseline characteristics of post-AMI patients

In this matched nested case-control study, 114 post-AMI patients were included for each category of LV remodeling: adverse LV remodeling versus reverse LV remodeling (**Figure 1**). As shown in **Table 1**, the mean age of the 228 post-AMI patients was 54 years and 94.7% (n = 216) were male. There were no significant differences in baseline variables between the patients with adverse LV remodeling and patients with reverse LV remodeling, except for those on warfarin treatment (12 out of 114 vs 2 out of 114; p = 0.006), beta-blocker (85 out of 114 vs 103 out of 114; p = 0.002), and the expected echocardiographic changes after 6 months post-AMI (p < 0.001).

**Table 1:** Baseline characteristics of the Post-AMI patients.

	Post-AMI			
	Adverse LV Remodeling	Reverse LV Remodeling	*p*-value		
	n = 114	n = 114			
**Demographic**					
Mean age (years, mean (sd))	54 (8.5)	54 (8.5)	0.975		
Male n (%)	109 (95.6)	107 (93.9)	0.553		
Chinese n (%)	67 (58.8)	66 (57.9)	0.190		
Malay n (%)	27 (23.7)	18(15.8)			
Indian n (%)	16 (14.0)	27 (23.7)			
Other n (%)	4 (3.5)	3 (2.6)			
**Medical history**					
Diabetes n (%)	23 (20.4)	22 (19.3)	0.842		
Dyslipidemia n (%)	49 (43.0)	52 (45.6)	0.689		
Hypertension n (%)	49 (43.0)	47 (41.2)	0.788		
**Smoking Status**					
Non-Smoker n (%)	36 (31.6)	37 (32.5)	0.860		
Current Smoker n (%)	68 (59.6)	65 (57.0)			
Ex-Smoker n (%)	10 (8.8)	12 (10.5)			
**Lipid levels at baseline**					
Total cholesterol (mg/dL, mean (sd))	5.38 (1.3)	5.44 (1.3)	0.770		
HDL cholesterol (mg/dL, mean (sd))	1.08 (0.2)	1.19 (0.8)	0.161		
LDL cholesterol (mg/dL, mean (sd))	3.51 (1.2)	3.50 (1.2)	0.992		
Triglycerides (mg/dL, mean (sd))	1.89 (1.0)	2.15 (3.3)	0.421		
**Diagnoses**					
STEMI (%)	96 (84.2)	90 (78.9)	0.305		
NSTEMI (%)	18 (15.8)	24 (21.1)			
**Medications**					
Aspirin (%)	110 (96.5)	112 (98.2)	0.408		
P2Y12 inhibitor (%)	110 (96.5)	113 (99.1)	0.175		
Statin (%)	112 (98.2)	111 (97.4)	0.651		
Warfarin (%)	12 (10.5)	2 (1.8)	0.006		
ACE inhibitor (%)	64 (56.1)	75 (65.8)	0.135		
Beta-blocker (%)	85 (74.6)	103 (90.4)	0.002		
**Echocardiographic**					
EF at baseline (%, median (95% CI)	52.87 (50.18-54.87	49.91 (48.00-50.96)	0.052		
EF at 6 months (%, median (95% CI)	50.36 (45.46-53.07	58.10 (56.55-60.62)	< 0.001		
Change in LVEDV (%, median (95% CI) after 6 months	28.80 (24.34-34.70)	-11.76 (-16.21–9.86)	< 0.001		
Change in LVESV (%, median (95% CI) after 6 months	29.64 (27.32-31.69)	-25.89 (-27.74-24.24)	< 0.001		
Change in EF (%, median (95% CI) after 6 months	-2.70 (-5.60–0.45)	15.70 (11.58-20.30)	< 0.001		

Continuous data are presented as mean ± SD, or median with 95% Confidence Interval (CI). Categorical variables are presented as %. HDL, high density lipoprotein; LDL, low density lipoprotein; STEMI, ST-elevation myocardial infarction; NSTEMI, non-ST-elevation myocardial infarction; LVEDV, left ventricular end diastolic volume; LVESV, left ventricular end systolic volume; EF, ejection fraction; sd, standard deviation.

### Temporal trends of plasma TF activity in post-AMI patients

**Figure 2:**
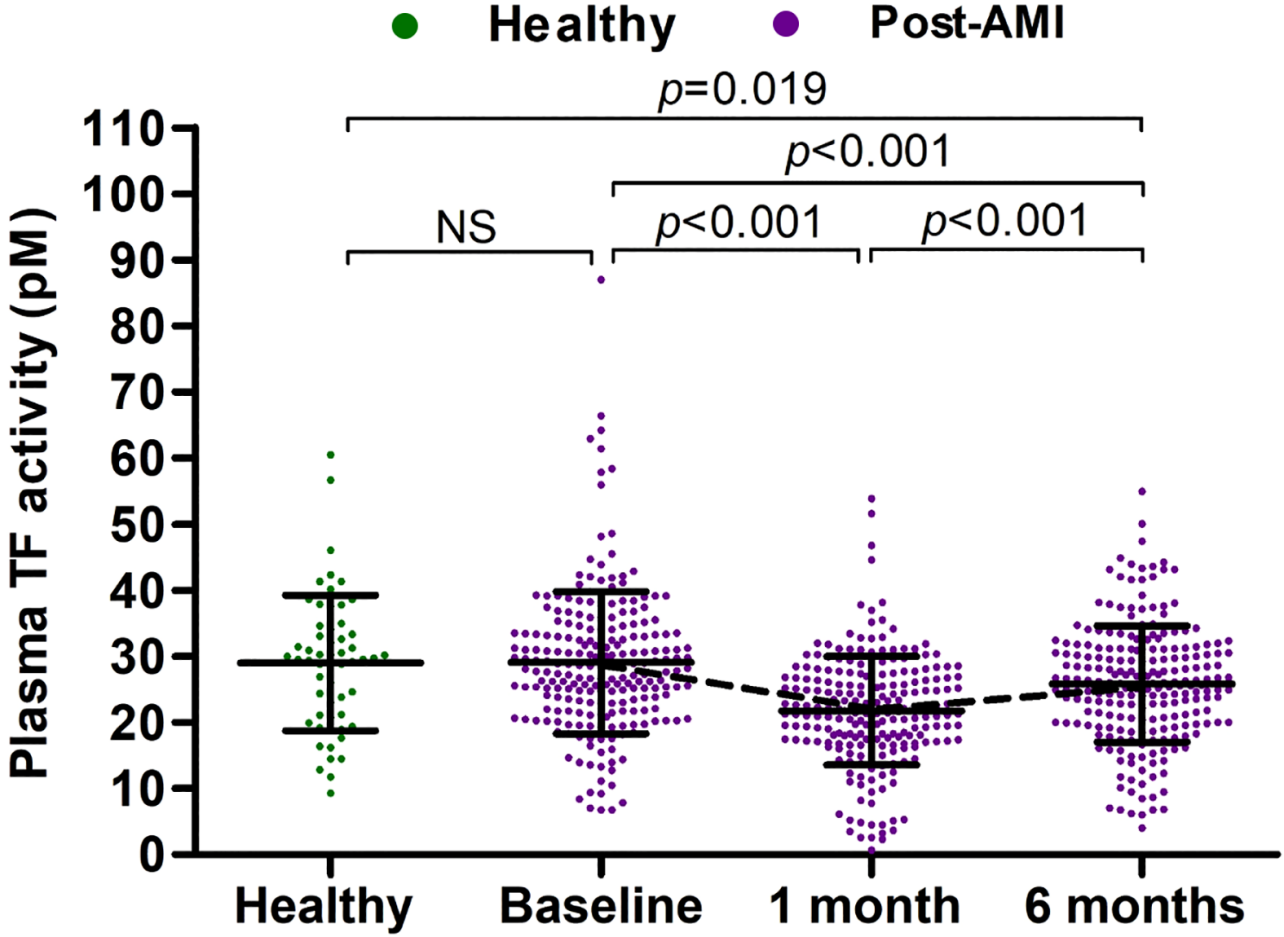
Plasma TF activity in healthy subjects and post-AMI patients. Plasma TF activity in healthy subjects (n = 57) and post-AMI patients (n = 228) was presented as mean ± SD and statistically analyzed with unpaired and paired t-tests. NS, no significance.

As shown in **Table S1** and **Figure 2**, the baseline (within 72 hours post PCI for AMI patients) levels of plasma TF activity in post-AMI patients were comparable to plasma TF activity in healthy subjects. Compared to baseline, plasma TF activity in patients was lower at 1 month (21.78 ± 8.23 vs 29.05 ± 10.75 pM; p<0.001) and 6 months (25.84 ± 8.80 vs 29.05 ± 10.75 pM; p<0.001) post-AMI. TF activity in patients partially recovered at 6 months post-AMI although it remained lower than baseline levels and that in healthy subjects.

**Figure 2**. Plasma TF activity in healthy subjects and post-AMI patients.

### Plasma TF activity in patients with adverse versus reverse post-AMI LV remodeling

**Figure 3:**
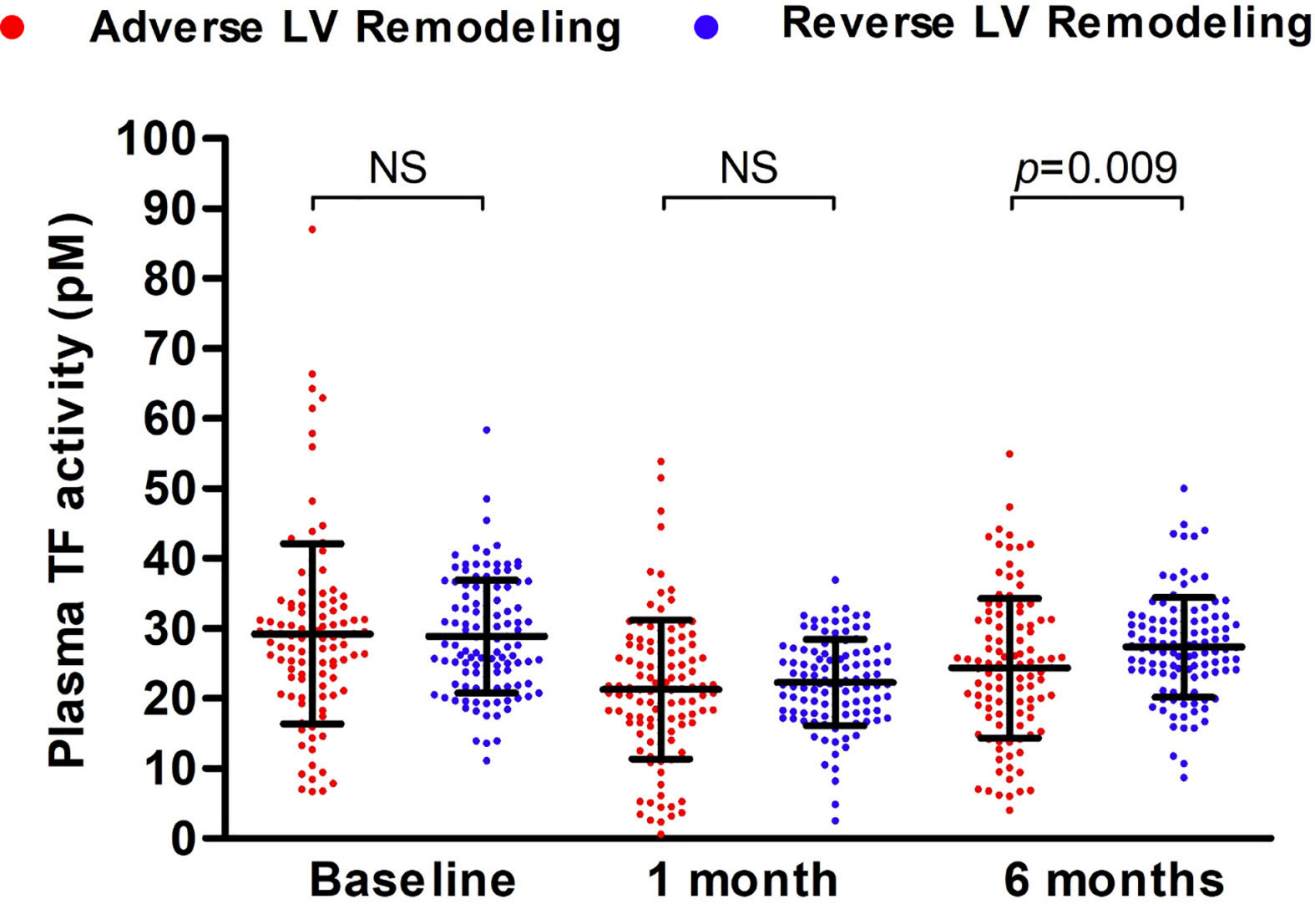
Plasma TF activity in patients with adverse versus reverse LV remodeling during 6-months follow-up. Plasma TF activity of patients with post-AMI adverse (n = 114) or reverse (n = 114) LV remodeling was presented as mean ± SD and statistically analyzed with unpaired t-test. NS, no significance.

TF activity decreased at 1 month and partially recovered at 6 months post-AMI in both patients with adverse LV remodeling and those with reverse LV remodeling (**Figure 3**). However, plasma TF activity recovered more at 6 months in patients with reverse LV remodeling than that in patients with adverse LV remodeling (27.35 ± 7.14 vs 24.34 ± 9.99; p=0.009). Plasma TF activity at 6 months post-AMI differed between those with adverse versus reverse LV remodeling (OR 0.960, 95% CI: 0.931-0.991; p=0.011) independent of cardiovascular risk factors, including age, sex, ethnicity, hypertension, dyslipidaemia, diabetes mellitus and smoking.

### Clinical correlates of plasma TF activity

The clinical correlations of plasma TF activity in post-AMI patients were identified using the ML-SEM model (**Table 2**). Out of a total of 15 clinical variables, Indian ethnicity, current smoking, and prescription of P2Y12 inhibitors were associated with higher plasma TF activity while warfarin treatment was associated with lower plasma TF activity. Other variables including age, gender, diabetes, dyslipidemia, hypertension, total cholesterol, HDL cholesterol, LDL cholesterol, triglycerides, aspirin, statin, beta-blockers and ACE inhibitors were not significantly correlated with plasma TF activity.

**Table 2:** ML-SEM modeling for plasma TF activity in post-AMI patients.

	TF activity		
	Coefficient	p-value	95% Cl
**Demographic**			
Age	0.007	0.883	-0.084 – 0.098
Female	2.692	0.101	-0.526 – 5.911
Chinese	Ref	Ref	Ref
Malay	0.956	0.315	-0.910 – 2.823
Indian	**2.446**	**0.010**	**0.578 – 4.313**
Other	3.819	0.106	-0.813 – 8.450
**Smoking Status**			
Non-Smoker	Ref	Ref	Ref
Current Smoker	**1.763**	**0.033**	**0.140 – 3.385**
Ex-Smoker	-0.386	0.777	-3.067 – 2.294
**Medical History**			
Diabetes	-0.298	0.770	-2.302 – 1.705
Dyslipidemia	-0.161	0.842	-1.739 – 1.418
Hypertension	0.442	0.599	-1.206 – 2.089
**Lipid levels at baseline**			
Total cholesterol	-0.091	0.909	-1.648 – 1.467
HDL cholesterol	0.861	0.601	-2.365 – 4.088
LDL Cholesterol	0.184	0.832	-1.521 – 1.890
Triglycerides	-0.037	0.849	-0.424 – 0.349
**Medication**			
Aspirin	4.846	0.076	-0.500 – 10.192
P2Y12 inhibitor	**8.938**	**0.005**	**2.726 – 15.151**
Statin	-1.986	0.530	-8.183 – 4.211
Warfarin	**-13.819**	**< 0.001**	**-17.403 – -10.236**
Beta-blockers	-0.182	0.906	-3.221 – 2.856
ACE Inhibitors	1.025	0.395	-1.349 – 3.399

Bolded values are those with p-value <0.05.

## Discussion

TF has been associated with AMI occurrence and post-AMI adverse events including mortality (18–23). Although the involvement of TF in AMI and post-AMI LV remodeling was reported in preclinical animal models (9, 27), the contribution of TF in post-AMI LV remodeling in human patients remains unclear. In this study, temporal changes of plasma TF activity showed a stronger recovery after the early post-MI fall of plasma TF activity among patients with reverse LV remodeling, independent of gender, age and relevant cardiovascular risk factors.

Studies on the role of plasma TF in AMI have produced conflicting results. While elevation in plasma TF levels were observed in patients with ACS or AMI (17, 20–22, 28–31), Roldan et al. reported no difference between AMI patients and healthy subjects (24). The discrepancy among those studies may be largely due to differences in experimental design and the timing of blood samples. Recently we found that myocardial TF expression decreased following AMI but partly recovered during chronic phase of LV remodeling in an animal model (9). Accordingly, we now demonstrate that plasma TF activity in post-AMI patients follows a similar V-shaped temporal pattern over 6-month follow-up. Similar dynamic change of plasma TF activity in post-AMI patients was also observed by Sambola et al. (22). These findings suggest that the sampling time point could significantly confound TF levels. Previous studies have reported higher plasma TF levels in AMI patients than in healthy controls in samples collected shortly after onset of AMI (prior to PCI) (17, 19, 21) with no difference reported in samples collected 2-3 months after AMI (24). Standard interventions such as PCI and medications including heparin, may partly account for the similar plasma levels between healthy controls and post-AMI patients within 3 days after PCI in the current study or 2-3 months after PCI as reported by Roldan et al. (24). Heparin has been shown to induce synthesis and secretion of TF pathway inhibitor (TFPI), a potent inhibitor of the extrinsic coagulation cascade by inactivating coagulation factor Xa and TF-factor VIIa (32, 33). As such, heparin administration may mask increases in plasma TF activity in the acute phase of AMI (“baseline” in our study).

The V-shaped dynamic change in plasma TF activity during the 6-month follow-up after AMI is unexpected. The initial decrease in plasma TF activity at 1-month post-AMI may be partly caused by anticoagulant medications. Apart from early use of heparin as discussed above, the majority of patients (> 97%) in our study were treated with dual antiplatelet therapy, aspirin and P2Y12 inhibitors (clopidogrel, ticagrelor or prasugrel) over the post MI period. Activated platelets release extracellular vesicles that have been identified as a major source of plasma TF (34). P2Y12 inhibitors such as Ticagrelor suppress release of procoagulant extracellular vesicles from platelets (35). However, antiplatelet treatment appeared associated with higher plasma TF activity in post-AMI patients. This discrepancy might be caused by different dosage or duration of antiplatelet treatment during follow-up, and/or some unaware confounding factors. Unfortunately, we have limited information on antiplatelet treatment drug dosage, duration or compliance of individual patients in this study. Aspirin therapy may reduce blood counts of TF-bearing extracellular vesicles of monocyte and smooth muscle cell origin (36). On the other hand, smoking confers a hypercoagulable state and has been shown to upregulate monocyte TF in patients with metabolic syndrome (37) and is associated with higher plasma TF activity in post-AMI patients in the current study. Of note, monocyte-derived TF-bearing extracellular vesicles account for the second largest pool of thrombogenic extracellular vesicles after platelet-derived extracellular vesicles (38–40). Statins, which were prescribed in 98% of post-AMI patients in the current study, have also been reported to reduce blood counts of TF-bearing extracellular vesicles (11, 41). In addition, Warfarin, may contribute to lower plasma TF activity (42, 43) during follow-up even though only 6% patients took it. Besides anticoagulant medications, consumption of TF or TF-bearing extracellular vesicles during thrombus formation and wound healing (44–46) following AMI or PCI-induced endothelial injury may reduce the circulating active TF levels. After 6 months post PCI, there should be no more such consumption of TF thereby allowing recovery of circulating concentrations. Early cessation of heparin therapy and possible reduction in patient adherence to oral anticoagulant medications during follow-up may partly account for the restoration of plasma TF activity at 6 months post-AMI. Although ACE inhibitors have been reported to reduce TF expression (12), their usage is not associated with plasma TF activity in post-AMI patients in the current study. This is likely because TF antigen expression in vascular cells does not always correlate well with TF activity (47). Moreover, beta-blocker was used in more patients with reverse LV remodeling, however, it did not exhibit obvious influence or confounding effects on plasma TF activity in our study (**Table 2**). Nevertheless, we observed that the dynamic change in plasma TF activity during post-AMI follow-up differed between reverse and adverse LV remodeling independent of other variables. Further investigation to unravel the underpinning mechanisms and their biological significance is warranted.

Several limitations of this study should be noted. Limitations include lack of blood collections at admission to enable capture of the full temporal profile of plasma TF activity from prior to PCI and introduction of pharmacotherapies. In addition, most patients were male in the study and healthy controls were younger than post-AMI patients. The tissue origins of TF contributing to plasma TF activity in post-AMI patients remains unclear and is beyond the scope of the current study.

## Conclusions

In summary, we demonstrate that plasma TF activity in post-AMI patients presents a V-shaped dynamic change over 6 months following PCI treatment - with a stronger restoration of plasma TF activity in patients exhibiting reverse LV remodeling during the 6 months follow-up period.

## Data availability statement

The raw data supporting the conclusions of this article will be made available by the authors, without undue reservation.

## Ethics statement

The studies involving human participants were reviewed and approved by National University of Singapore Institutional Review board (NHG DSRB Ref: 2015/01156). The patients/participants provided their written informed consent to participate in this study.

## Author contributions

JWW designed the experiments. XCL, SMJMY, SYC, XW and SPC performed experiments and data analysis. SHT, XY and MYC provided materials. SPC, AMR, CJC, MYC, and JWW contributed to data interpretation. XCL, SMJMY and JWW wrote the manuscript. All authors contributed to the article and approved the submitted version.

## Funding

This work was supported by the National University Health System collaborative grant (NUHS O-CRG 2016 Oct-23) to JWW and MYC; and the National Medical Research Council Centre Grant (NMRC CG21APR1008) to AMR, MYC and JWW; SYC would like to thank the generous support by the ESR and Loo Geok Eng Foundation PhD scholarship.

## Conflict of interest

The authors declare that the research was conducted in the absence of any commercial or financial relationships that could be construed as a potential conflict of interest.

## Publisher’s note

All claims expressed in this article are solely those of the authors and do not necessarily represent those of their affiliated organizations, or those of the publisher, the editors and the reviewers. Any product that may be evaluated in this article, or claim that may be made by its manufacturer, is not guaranteed or endorsed by the publisher.
