# Supplementary material for: Plasma tissue factor coagulation activity in post-acute myocardial infarction patients
Source: Front Endocrinol (Lausanne). 2022 Sep 23;13:1008329. doi: 10.3389/fendo.2022.1008329 (PMC9540383; doi:10.3389/fendo.2022.1008329)
Supplement: Supplementary file 1 [file Table_1.docx]

**Supplemental Table S1.** Plasma TF activity in post-AMI patients and healthy subjects

| TF activity (pM) | Healthy Subjects  n = 57 |  | Post-AMI | | |
| --- | --- | --- | --- | --- | --- |
|  |  |  | Total Post-AMI  n = 228 | Adverse LV Remodeling  n =114 | Reverse LV Remodeling  n = 114 |
| Baseline | 29.01 (10.22) |  | 29.05 (10.75) | 29.24 (12.89) | 28.87 (8.11) |
| 1 month |  |  | 21.78 (8.23) | 21.29 (9.89) | 22.28 (6.15) |
| 6 months |  |  | 25.84 (8.80) | 24.34 (9.99) | 27.35 (7.14) |
